# Deciphering the comprehensive relationship between 5′ UTR and 3′ UTR sequences with deep learning

**DOI:** 10.1093/bioinformatics/btag634

**Published:** 2026-08-26

**Authors:** Kanta Suga, Keisuke Yamada, Michiaki Hamada

**Affiliations:** Department of Electrical Engineering and Bioscience, Graduate School of Advanced Science and Engineering, Waseda University, Tokyo 169-8555, Japan; Department of Electrical Engineering and Bioscience, Graduate School of Advanced Science and Engineering, Waseda University, Tokyo 169-8555, Japan; Department of Bioengineering, University of Pennsylvania, Philadelphia, PA 19104, United States; Department of Electrical Engineering and Bioscience, Graduate School of Advanced Science and Engineering, Waseda University, Tokyo 169-8555, Japan; Cellular and Molecular Biotechnology Research Institute (CMB), National Institute of Advanced Industrial Science and Technology, Tokyo 169-8555, Japan; Graduate School of Medicine, Nippon Medical School, Tokyo 113-8602, Japan

## Abstract

**Motivation:**

Recent advances in mRNA therapeutics have driven further research on the untranslated regions (UTRs) of mRNA. However, prior studies have mainly focused on either the 5′ or 3′ UTR individually. Increasing evidence suggests potential cooperative effects between these two regions, which remain largely unexplored in computational studies.

**Results:**

We present a deep learning-based approach to predicting relationships between 5′ and 3′ UTRs by leveraging latent representations from a pre-trained RNA language model and contrastive learning. Our method effectively identifies highly related UTRs, uncovering sequence and expression characteristics that suggest functional interplay. Our analysis revealed that Highly Related UTRs (HRUs) are significantly enriched in genes associated with neural development, exhibit distinctive UTR length and secondary structure characteristics, and are involved in cell type-specific regulation of translation efficiency. These findings provide new insights into UTR co-optimization for mRNA therapeutics.

**Availability:**

The source code is available for free at https://github.com/hmdlab/utr_pairpred.git. The data and intermediate files used in our analysis are available at https://waseda.box.com/v/utr-pairpred-data.

## 1 Introduction

Untranslated regions (UTRs) at the 5′ and 3′ ends of eukaryotic mRNAs are critical regulators of gene expression, profoundly influencing mRNA stability and translational efficiency. The 5′ UTR plays a crucial role in ribosome recruitment, while the 3′ UTR contains regulatory elements that control mRNA decay and translation. In mRNA-based therapeutics, these factors are particularly important, and a growing body of research highlights the significance of UTR sequences themselves in maximizing mRNA performance. For instance, Asrani *et al*. (2018) and Leppek *et al*. (2022) demonstrated that in various natural mRNAs, optimizing 5′-3′ UTR combinations leads to improved translation efficiency and stability. These findings emphasize that UTR sequences are the active determinants of mRNA’s therapeutic potential.

Recent engineering efforts of UTR sequences have focused on 5′ or 3′ UTR independently (Sample *et al*. 2019, Kirshina *et al*. 2023, Castillo-Hair *et al*. 2024, Fu *et al*. 2024, Liu *et al*. 2024), while naturally occurring UTRs act in coordination to regulate translation. In a well-established closed loop model, interactions between the cap-binding complex and poly(A)-binding proteins physically bridge the 5′ and 3′ ends of an mRNA, facilitating translation initiation (Tomek and Wollenhaupt 2012, Archer *et al*. 2015, Kim *et al*. 2023). Beyond this general mechanism, certain transcripts modulate translation levels via direct base-pairing interactions between their 5′ and 3′ UTRs (Guo *et al*. 2001, Chen and Kastan 2010, Ruiz de los Mozos *et al*. 2013, Braun *et al*. 2017). Therefore, for both basic science and engineering, it is crucial to understand the sequence features that regulate the interaction between 5′ and 3′ UTRs.

The increasing availability of large-scale genomic data has enabled us to thoroughly investigate 5′–3′ UTR relationships. With the widespread adoption of high-throughput techniques, such as RNA sequencing (RNA-seq) (Wang *et al*. 2009) and ribosome profiling (Ribo-seq) (Ingolia *et al*. 2009), the expansion of large RNA sequence databases provides a valuable resource for data-driven analysis (The-RNAcentral-Consortium 2017).

Recent advances in deep learning models for natural language processing have spurred significant research into RNA language models, representing a novel computational approach in bioinformatics (Chu *et al*. 2024, Yu *et al*. 2024, Zhang *et al*. 2024). RNA-FM (Chen *et al*. 2022) is one such language model, trained on millions of RNA sequences (The-RNAcentral-Consortium 2017). Embeddings generated by RNA-FM demonstrated superior predictive performance compared to conventional methods across diverse RNA-related tasks, such as mean ribosome loading prediction, protein-RNA interaction prediction and secondary structure prediction. In addition, RiNALMo (Penić *et al*. 2024), which was trained on a larger corpus of RNA sequences using a more sophisticated model architecture, is also a representative RNA language model and has demonstrated high performance across more diverse range of tasks, including splice-site prediction. This success suggests that RNA language models can capture more flexible and biologically meaningful embedding than traditional methods.

In parallel, contrastive learning (Chen *et al*. 2020) has emerged as a powerful technique for learning representations that associate information across different domains. A notable example comes from protein science, where proteins and their ligands (e.g. drugs) are embedded into a joint representation space. This protein–ligand co-embedding approach dramatically improved the prediction of molecular interactions (Singh *et al*. 2023, Wang *et al*. 2024, Zhang *et al*. 2025). Inspired by such approaches, a similar strategy can be applied to 5′ and 3′ UTRs to obtain meaningful joint representations.

Here, we propose a computational method to analyze the relationship of 5′ and 3′ UTR sequences in mRNA. While traditional sequence alignment and statistical approaches can identify explicit sequence motifs (Mignone *et al*. 2002, Xie *et al*. 2005, Huang *et al*. 2006), the underlying mechanisms of 5’–3’ UTR interactions remain largely unknown and may involve complex, non-linear relationships that are difficult to capture with conventional methods. Machine learning approaches are well-suited for detecting such latent patterns without requiring prior knowledge of the specific interaction mechanisms. Our primary objective is to identify UTR pairs that potentially interact, rather than to elucidate the precise molecular mechanisms underlying these interactions. By combining latent representations extracted from a pre-trained RNA language model (RNA-FM, RiNALMo) with contrastive learning (Chen *et al*. 2020), we enabled the model to distinguish UTR pairs derived from the same mRNAs from those that are not. Further analysis focusing on highly related UTRs (HRUs) revealed that specific combinations of UTRs may play a significant role in regulating translation levels and interactions with RNA binding proteins (RBPs) involved in mRNA processing. This study presents the novel computational method for comprehensively investigating the relationship between the 5′ and 3′ UTRs, providing an avenue to elucidate the synergistic regulatory roles of UTR interactions and to guide the rational design of advanced mRNA therapeutics.

## 2 Materials and methods

### 2.1 Data collection

In this study, we constructed datasets of untranslated region (UTR) sequences by extracting GENCODE protein-coding transcripts (Frankish *et al*. 2023) from human (gencode.v44) and mouse (gencode.vM33) genomes. GENCODE is widely recognized as one of the most comprehensive and reliable genome annotation resources, serving as the standard reference for human and mouse gene annotations. We selected transcripts that contained both 5′ and 3′ UTRs, each exceeding 10 nucleotides in length. For genes with multiple variants, only the longest transcript was retained. Redundant sequences were removed using CD-HIT (Li and Godzik 2006) with a sequence similarity threshold of 0.9, and this filtering was applied, respectively, to 5′ and 3′ UTR sets. The final dataset comprised 16 819 transcripts for human and 18 165 for mouse.

### 2.2 Sequence embedding with RNA language model

The UTRs of endogenous mRNAs vary greatly in length, ranging from hundreds to thousands of nucleotides. Due to the length diversity, directly inputting the entire nucleotide sequence into a model is challenging. To address this challenge, we converted UTR sequences into fixed-dimensional embeddings using an RNA language model and provided these embeddings as input to the prediction model.

We employed two state-of-the-art pre-trained models designed for various RNA-related tasks: RNA-FM (hidden dimension = 640) and RiNALMo (hidden dimension = 1280). RNA-FM is a Transformer encoder-based model with 100 million parameters, trained on 23.7 million ncRNA sequences from the RNAcentral database (The-RNAcentral-Consortium 2017). On the other hand, RiNALMo was trained on a larger dataset of 36 million RNA sequences from RNAcentral and other databases such as Rfam and Ensembl. RiNALMo has a BERT-style architecture with 650 million parameters, incorporating advanced techniques such as rotary positional embeddings (RoPE) (Su *et al*. 2024), SwiGLU activation functions (Shazeer 2020), and FlashAttention2 (Dao 2023). To obtain embeddings using RNA-FM, we extracted the embedding corresponding to the [CLS] token from the model’s output for a given input sequence. For UTR sequences exceeding the model’s maximum input length (1022 nucleotides), the sequence was segmented at this length, [CLS] token embeddings were obtained for each segment, and mean pooling was applied to generate the final embedding (e.g. a sequence of 2050 nt was divided into segments of 1022, 1022, and 6 nucleotides, and embeddings from each segment were then combined through mean pooling). In contrast to RNA-FM, RiNALMo employs relative positional embeddings, and it does not have a maximum input length constraint. Therefore, we obtained a *D*-dimensional embedding from RiNALMo by mean pooling the L×D output embedding matrix along the *L* dimension, where *L* represents sequence length.

### 2.3 Data pair construction for prediction tasks

**Figure 1:**
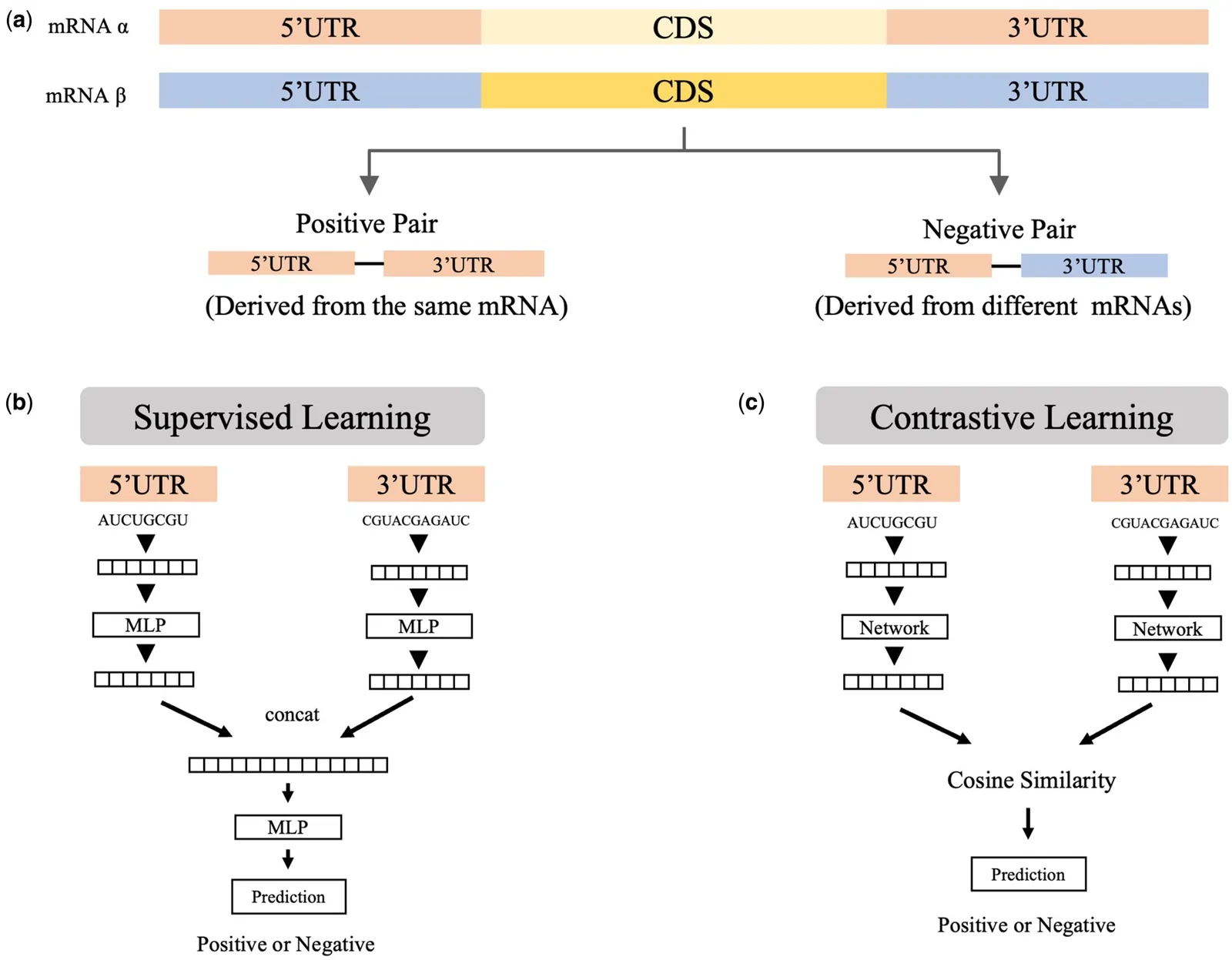
(a) Data construction for the UTR pair prediction task. Pairs of 5′ UTR and 3′ UTR sequences derived from the same mRNA were designated as positive pairs, while those from different mRNAs were defined as negative pairs. The prediction task aims to classify a given UTR pair as positive or negative with either (b) Supervised learning or (c) Contrastive learning method. In both methods, sequence embeddings extracted from a pre-trained RNA language model were used as the inputs for the prediction task.

For our prediction task, we constructed positive and negative UTR pairs as illustrated in Fig. 1a. Positive pairs consisted of 5′ UTR and 3′ UTR sequences derived from the same mRNA transcript, representing naturally co-occurring UTR combinations. Negative pairs were created by pairing 5′ UTR sequences with 3′ UTR sequences from different mRNA transcripts, representing non-native combinations. This paired dataset construction allowed us to train models that could distinguish between biologically relevant and random UTR associations.

### 2.4 Training methods

We employed two independent approaches–supervised learning and contrastive learning–to predict the relationship between 5′ and 3′ UTRs.

#### 2.4.1 Supervised learning

During supervised learning, the model was trained using explicitly labeled data (Fig. 1b). The 5′ and 3′ UTR sequences were passed through separate multilayer perceptron (MLP) layers, and their outputs were concatenated into a single vector. This concatenated vector was then reduced in dimensionality through three additional MLP layers, producing logits for prediction.

A target label of 1 was assigned to the pairs of 5′ and 3′ UTRs originating from the same mRNA, and 0 otherwise. The model was trained using binary cross-entropy loss to learn the correspondence between UTR pairs and their labels. After hyperparameter tuning, we set the batch size to 32, the learning rate to 1e-4, and used the Adam optimizer. To mitigate overfitting, we applied a dropout rate of 0.5.

#### 2.4.2 Contrastive learning

We also adapted a contrastive learning approach (Chen *et al*. 2020) to predict UTR pair relationships. In this approach, multiple positive pairs of 5′ UTR and 3′ UTR sequences from the same mRNA were collected into a batch. Within a batch, the model was trained to bring the representations of positive 5′–3′ UTR pairs closer together while pushing apart the representations of non-matching UTR pairs.

In contrastive learning, the similarity between UTR pairs was measured using the cosine similarity between their vector representations. Cosine similarity is a widely adopted metric in representation learning and natural language processing for measuring the similarity between high-dimensional embedding vectors (Mikolov *et al*. 2013, Radford *et al*. 2021). Unlike Euclidean distance, cosine similarity focuses on the directional alignment of vectors rather than their magnitude, making it particularly suitable for comparing learned representations where the relative orientation captures semantic relationships. During testing, we calculated the cosine similarity for a given 5′–3′ UTR pair and applied a similarity threshold (e.g. 0.0) to determine whether the pair originated from the same mRNA.

We treated the cosine similarity between the representations of 5′ and 3′ UTRs from contrastive learning as a quantitative measure of their relation score. By performing 10-fold cross-validation, we computed this cosine similarity for all mRNAs in the dataset. Hereafter, we refer to it as the relation score of the UTR pair (Fig. 1c).

#### 2.4.3 Random forest

As a baseline for performance comparison with the proposed method, we employed a random forest classifier (Breiman 2001). Similarly, the random forest model predicted whether a given pair of 5′ and 3′ UTR sequences originated from the same mRNA. Following the approach of Cao *et al*. (2021), we extracted various features from the sequences, such as length, GC content, energy, and k-mer frequency, and provided a total of 5450 features as input to the model.

### 2.5 Analysis for highly related UTRs

To elucidate biological factors contributing to the highly related UTRs, we conducted analysis from multiple perspectives, involving the sequence features of UTRs (2.5.1), mRNA-RBP interactions (2.5.2) and translation regulation (2.5.3).

#### 2.5.1 Length & MFE comparison

We performed a comparative analysis of the minimum free energy (MFE). MFE is an important indicator for the structural functionality of RNA sequence. As illustrated in Fig. 3a, the UTR regions were divided into subclasses for detailed comparison. In the case of the 5′ UTR, we compared the first 100 nucleotides (designated as 5′ UTR head) and the last 100 nucleotides (5′ UTR tail), as well as a 200-nucleotide region comprising the last 100 nucleotides of the 5′ UTR and the first 100 nucleotides of the CDS (designated as 5′ UTR_tail_cds). Because the 3′ UTR sequences are generally longer, we calculated the MFE for the first and last 500 nucleotides (designated as 3′ UTR head and 3′ UTR tail, respectively) and for a 600-nucleotide region formed by the junction of the last 100 nucleotides of the CDS with the beginning of the 3′ UTR (designated as 3′ UTR_head_cds). MFE calculations were performed using RNAfold Lorenz *et al*. (2011).

#### 2.5.2 Features of RBP interactions

**Figure 2:**
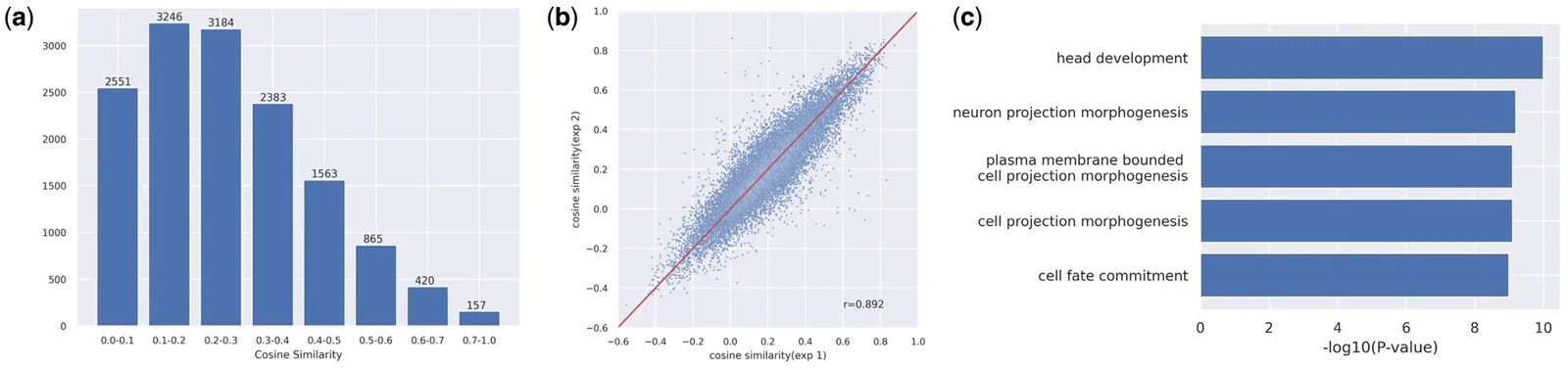
(a) Distribution of cosine similarity values for pairs of 5′ and 3′ UTRs of human mRNAs obtained using contrastive learning combined with RiNALMo embedding. The subset with the highest similarity range (0.7 ∼ 1.0; *n* = 157) was defined as “Highly Related UTRs (HRUs)” for further analysis. (b) Cosine similarity results obtained by repeating the contrastive learning + RiNALMo approach with different random seeds. The strong correlation observed across experiments indicates that the results used for the analysis are not coincidental. (c) The top five Gene Ontology terms with the highest –log10(*P* value) from the gene enrichment analysis of HRUs.

To investigate the association between HRUs and mRNA-RBP interactions, we conducted comprehensive analysis using eCLIP (Van Nostrand *et al*. 2016) data. eCLIP is a transcriptome-wide sequencing method to map RNA regions that interact with RBPs. On the mRNA side, we categorized transcripts into groups based on relation score thresholds, following the approach shown in Fig. 2(a). On the RBP side, we collected all available human eCLIP BED files from the ENCODE database (Moore *et al*. 2020). We calculated the number of eCLIP peak intersecting with the UTRs using BEDTools (Quinlan and Hall 2010). Subsequently, the mean number of peaks was computed within each UTR relation score group. These scores were individually calculated for 5′ and 3′ UTRs. To identify RBPs that preferentially bind to HRUs, we computed the ratio of the average intersection values between the HRUs (0.7–1.0) and the lowest relation score group (0.0–0.1). We then focused on the top 30 RBPs with the highest ratios for the downstream gene ontology analysis.

#### 2.5.3 Translation efficiency

To assess differences in translation efficiency between HRUs and the all other mRNA, we calculated translation efficiency (TE) across multiple human cell types and tissues. TE was calculated as


(1) $$TE=\frac{\text{Ribo-seq}(\text{TPM})}{\text{RNA-seq}(\text{TPM})}.$$

following the approach by Chothani *et al*. (2019). Raw RNA-seq and Ribo-seq reads were obtained from The Sequence Read Archive (Leinonen *et al*. 2011) and mapped using Bowtie 2 (Langmead and Salzberg 2012). The detailed data information is provided in Table S1. For statistical analysis, the Mann-Whitney U test was used to assess differences in TE distributions between HRUs and all other transcripts.

#### 2.5.4 In-silico mutagenesis by sliding-window randomization

Our prediction task demonstrated that the model jointly considers both the 5′ and 3′ UTRs when scoring their relationship. To resolve *which* sequence regions drive the predicted relationship, we performed a position-resolved in-silico mutagenesis analysis, in which local segments of a UTR are perturbed and the resulting change in the relation score is measured. This analysis was conducted on the human dataset using the best-performing model (contrastive learning with RiNALMo embeddings).

For each HRU transcript, we slid a window of length equal to 20% of the UTR length (stride of 5% of the length) along the 5′ and 3′ UTRs independently. The nucleotides within each window were randomized while the partner UTR was kept intact, and the UTR pair was re-embedded with RiNALMo and re-scored exactly as in the prediction pipeline. We used three randomization schemes that progressively preserve sequence properties: (i) *uniform*, in which window nucleotides are drawn independently and uniformly, destroying both base composition and nucleotide order; (ii) *shuffle*, a random permutation of the window that preserves base composition while destroying order; and (iii) *dinucleotide*, a dinucleotide-frequency-preserving shuffle that additionally preserves the local base-pairing propensity underlying RNA secondary structure, as the nearest-neighbor stacking free energies that govern duplex stability are parameterized at the dinucleotide level (Turner and Mathews 2010, Lorenz *et al*. 2011). Each window was randomized over 10 independent trials and the resulting relation scores were averaged. We defined the sensitivity of a window as Δ= (baseline relation score) −(mean randomized relation score); a large positive Δ indicates that the window is important for the predicted 5′–3′ UTR relationship. For each transcript, the *maximum-sensitivity window* was defined as the window with the largest *uniform* Δ across both UTRs.

To distinguish composition-driven from structure-driven sensitivity, we additionally re-scored each maximum-sensitivity window under the *shuffle* and *dinucleotide* schemes and partitioned its Δ into base-composition, base-pairing (secondary-structure) and single-stranded-order contributions; because the three schemes preserve a nested set of sequence properties, the difference in Δ between adjacent schemes isolates the contribution of the property that distinguishes them. The rationale, formulae and per-transcript values of this decomposition are given in Supplementary Note S1 and Table S3. As control experiment, we applied the identical procedure to 157 transcripts sampled from the lowest relation-score bin (0.0–0.1) and compared HRU and control sensitivity within ten equal-width relative-position bins of each UTR using the Mann–Whitney U test with Benjamini–Hochberg correction. Finally, the sequence of each maximum-sensitivity window was scanned against literature-consensus RNA structural signatures and RBP-binding motifs encoded as regular expressions, including RNA G-quadruplexes (rG4), AU-rich elements (AUUUA), polypyrimidine tracts, and the m^6^A consensus (DRACH).

## 3 Results

### 3.1 Performance comparison

For the task of predicting the correspondence between the 5′ and 3′ UTR on the same mRNA, we evaluated four methods, each combining an RNA language model (RNA-FM or RiNALMo) with a different learning strategy (supervised learning or contrastive learning). Additionally, as a baseline, we implemented a random forest classifier with extracted sequence features and compared its performance. The results are presented in Table 1. RNA language model-based methods (RNA-FM and RiNALMo) significantly outperformed the random forest baseline. Both models showed enhanced performance with contrastive learning compared to supervised learning. Notably, RiNALMo, likely benefiting from its deeper architecture and higher-dimensional embeddings, consistently achieved the highest scores. These results suggest that contrastive learning with a large-scale RNA model is a promising approach for estimating relationships between 5′ and 3′ UTRs.

**Table 1:** Performance comparison of UTR pair prediction methods across different embedding and learning approaches.

Embedding method	Learning method	Human		Mouse	
		AUROC	AUPRC	AUROC	AUPRC
Extracted feature	Random Forest	0.551	0.523	0.520	0.493
RNA-FM	Supervised	0.698	0.677	0.714	0.698
	Contrastive	0.711	0.692	0.724	0.709
RiNALMo	Supervised	0.715	0.693	0.736	0.719
	Contrastive	**0.726**	**0.710**	**0.741**	**0.728**

Performance metrics for different UTR pair prediction approaches on human and mouse datasets. The models were evaluated using the area under the receiver operating characteristic curve (AUROC) and area under the precision-recall curve (AUPRC). The highest values in each column are highlighted in bold. All experiments were conducted using 10-fold cross-validation.

### 3.2 Analysis for highly related UTRs

We investigated the factors contributing to the relationship between untranslated regions (UTRs) on the same mRNA from multiple perspectives. For our analysis, we utilized the results obtained from the combination of contrastive learning and RiNALMo, which achieved the highest performance in the UTR pair prediction task. In the model trained with contrastive learning, cosine similarity was computed for each input pair of 5′ and 3′ UTRs. We defined this cosine similarity as a relation score between the two regions. A higher relation score may suggest a stronger functional association between the 5′ and 3′ UTR, indicating that their co-occurrence on the same mRNA is biologically significant. Using 10-fold cross-validation, we obtained relation scores for all mRNAs in the human dataset; the distribution of these scores is shown in Fig. 2(a). Subsequent analyses focused on samples with the highest relation scores (0.7–1.0, *n* = 157), which we designated as *H*ighly *R*elated *U*TR**s** (HRUs). When we calculated the relation scores for all mRNAs in two independent experiments, a strong correlation was observed between experiments, confirming the reproducibility of these scores (Fig. 2b).

To investigate whether the genes associated with HRUs exhibit any distinctive characteristics, we performed a gene enrichment analysis using Metascape (Zhou *et al*. (2019)). The results revealed a significant enrichment of genes involved in developmental processes, particularly “head development” and “neuron projection morphogenesis,” suggesting that interactions between UTRs are involved in the translation regulation of those genes (Fig. 2c).

#### 3.2.1 Analysis for sequence features

**Figure 3:**
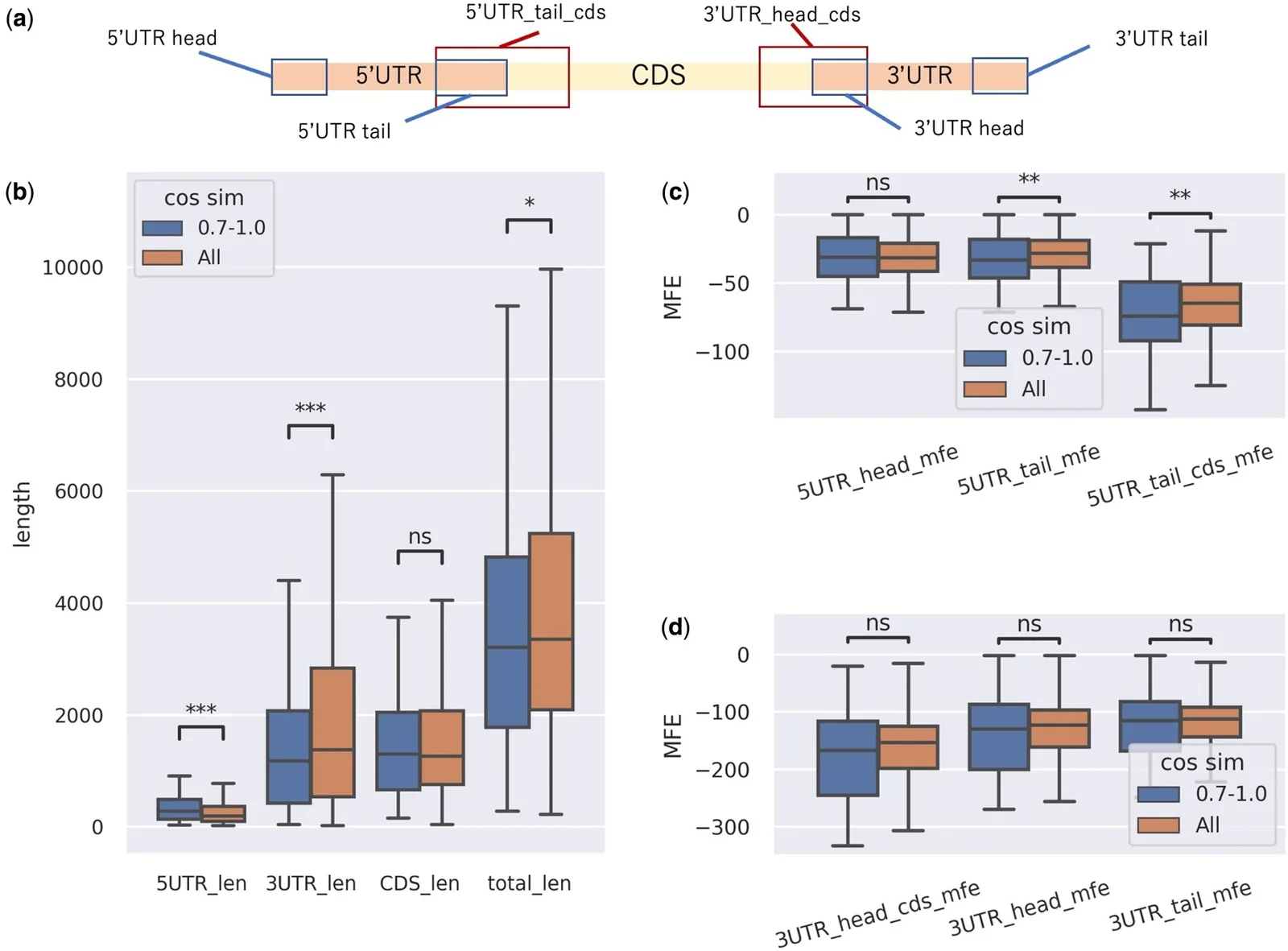
Sequence feature analysis. (a) The names of the subdivisions for each analyzed mRNA region. Comparison of each mRNA region between HRUs and all other transcripts about (b) sequence length, (c) MFE (kcal/mol) in each subdivision of the 5′ UTR, (d) MFE in each subdivision of the 3′ UTR.

We analyzed the sequence characteristics of HRU-containing mRNAs by dividing each UTR region into detailed subregions and analyzing their features (Fig. 3a). First, we compared the lengths of each region between HRU-containing mRNAs and all other mRNAs. This analysis revealed that, relative to the overall mRNA population, HRU-containing mRNAs exhibit a significantly longer 5′ UTR, a significantly shorter 3′ UTR, no significant difference in CDS length, and an overall significantly shorter transcript length (Fig. 3b).

To investigate whether HRUs exhibit distinctive structural characteristics, we compared the MFE values between mRNAs with HRUs and all other mRNAs in each subregions. The mRNAs with HRUs exhibited significantly lower MFE value at the end of 5′ UTR and at the intersection between 5′ UTR and CDS, suggesting that secondary structure formation in the 5′ UTR is more pronounced. Collectively, mRNAs with HRUs tend to have a longer, more structured 5′ UTR but a shorter 3′ UTR, resulting in a shorter overall transcript. These sequence and structural features are likely to influence various aspects of gene expression regulation, including mRNA stability and translation efficiency.

#### 3.2.2 Analysis for interactions with RBPs

**Figure 4:**
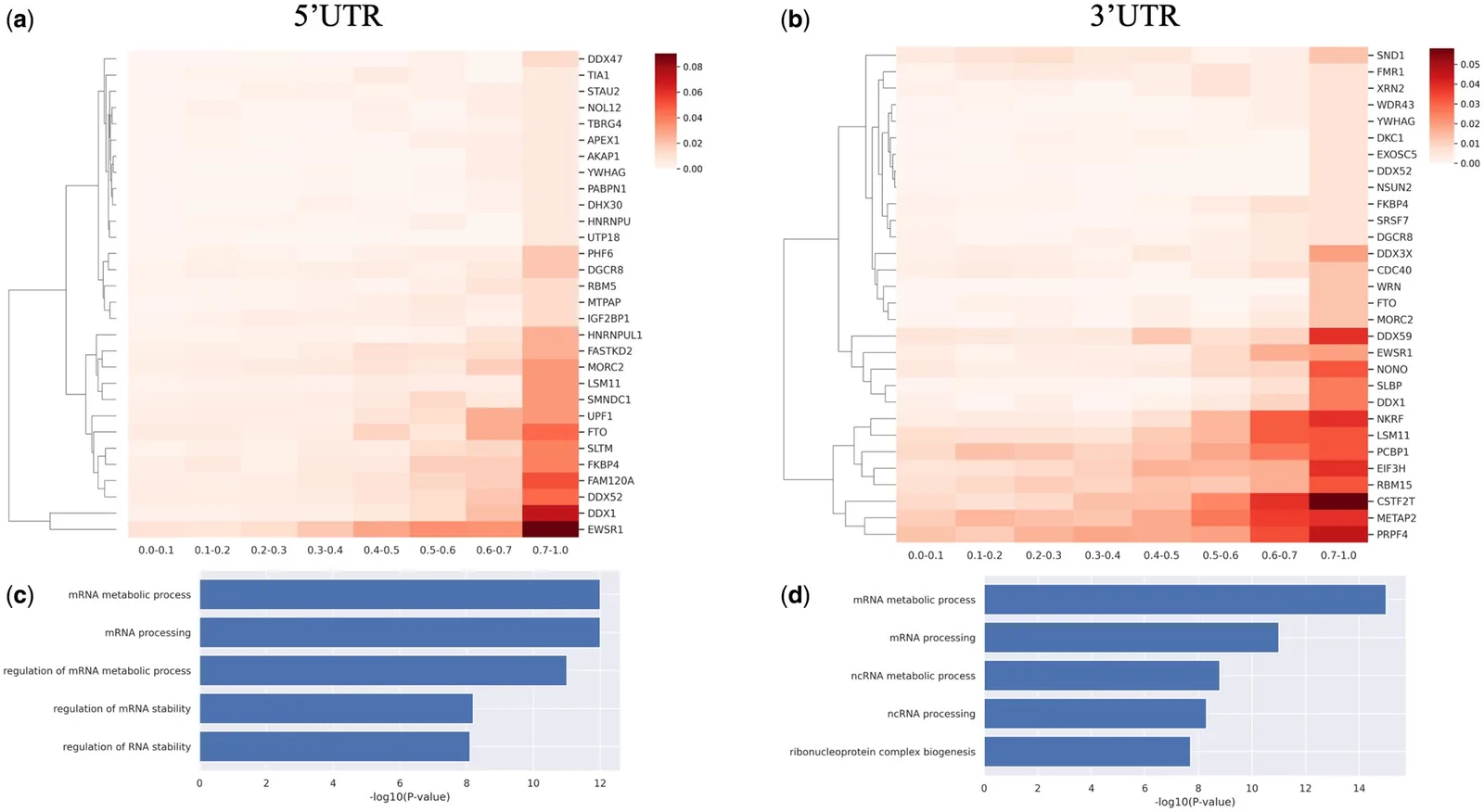
Analysis of interactions between 5′/3′ UTRs and RNA-binding proteins (RBPs). The intersection between all eCLIP datasets in ENCODE and the UTR regions was first calculated and averaged for each threshold of the relation score. By comparing the mean values of HRUs with those of the 0.0–0.1 relation score group, the top 30 RBPs were identified. Panels (a) and (b) show cluster heatmaps for these 30 RBPs in the 5′ and 3′ UTR, respectively, while panels (c) and (d) present the results of functional enrichment analysis for the 5′ and 3′ UTR, respectively.

RNA binding proteins (RBPs) are known to significantly influence RNA function through interactions with UTRs (Liu and Cao 2023). To investigate RBP-binding profiles in HRUs and other UTR pairs, we performed a cross-analysis between the UTRs and eCLIP data. Clustering analysis revealed a distinct group of RBPs that preferentially bind to HRUs in both the 5′ and 3′ UTRs (Fig. 4a and b). Subsequent gene enrichment analysis of these RBPs indicated significant enrichment in genes associated with mRNA metabolic processes and mRNA processing, suggesting a strong functional relevance of these interactions (Fig. 4c and d).

#### 3.2.3 Analysis for translation efficiency

**Figure 5:**
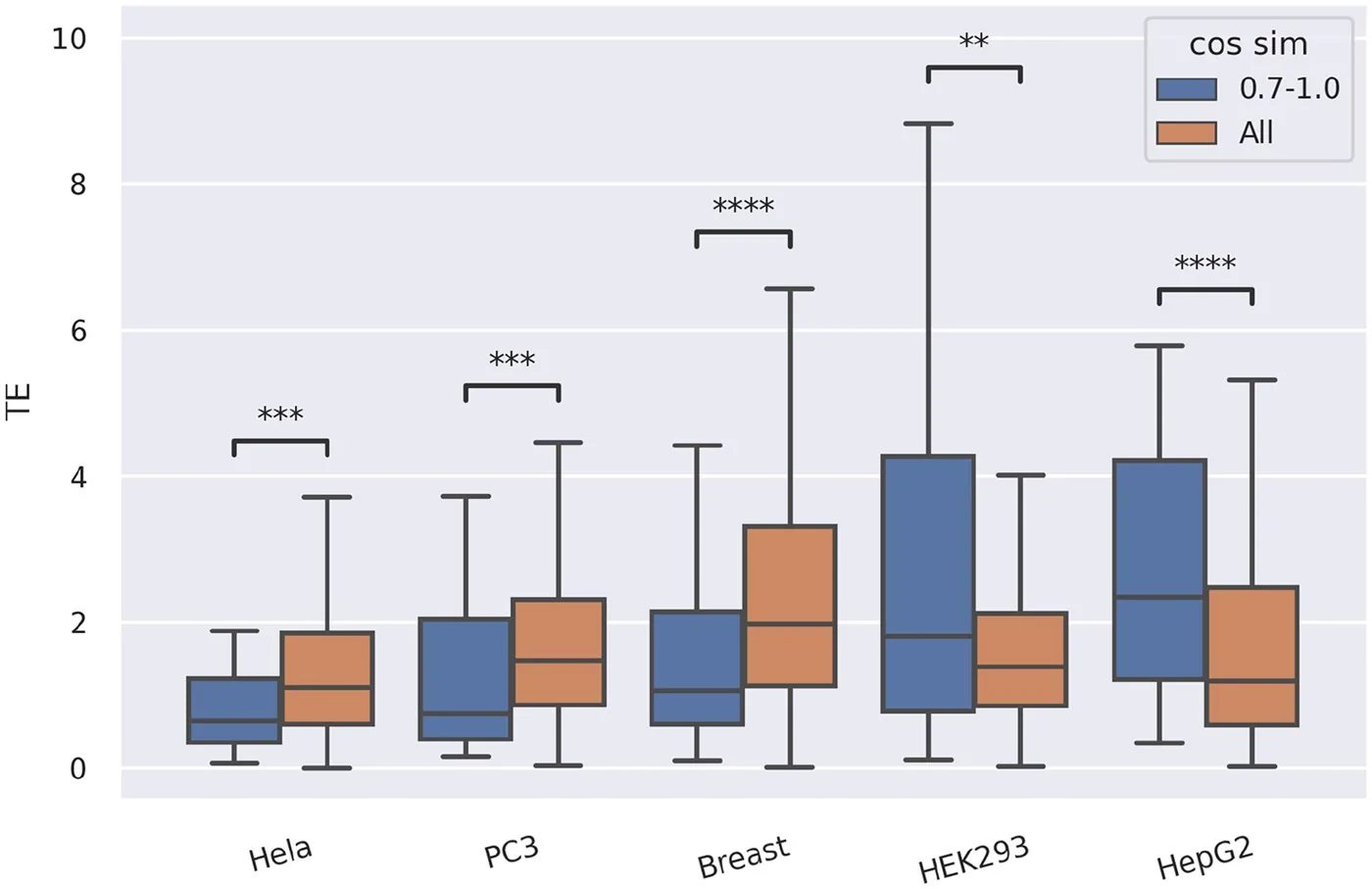
Comparison of translation efficiency (TE) between HRUs and other mRNAs across different cell lines by the Mann-Whitney *U* test. TE was analyzed separately for each cell line. In HepG2 and HEK293 cells, HRUs exhibited significantly higher TE, whereas in HeLa, PC3, and Breast cell lines, HRUs showed significantly lower TE.

The UTR sequence significantly regulates the mRNA translation levels. To investigate whether substantial differences in translation efficiency (TE) exist between HRUs and other mRNAs, we calculated and compared TEs across multiple cell types and tissues. Interestingly, as shown in Fig. 5, mRNAs containing HRUs exhibited significantly higher TE relative to the overall average in certain cell types (HepG2: $P=3.57\times 10^{-6}$, HEK293: $P=4.98\times 10^{-3}$), whereas they displayed significantly lower TE in others (Breast: $P=2.09\times 10^{-7}$, PC3: $P=3.21\times 10^{-4}$, HeLa: $P=3.39\times 10^{-4}$). In some other cell types, there was no significant difference in TE (Fig. S1, available as supplementary data at *Bioinformatics* online). These findings suggest that HRUs may contribute to the regulation of translation efficiency in a cell-line or tissue specific manner.

#### 3.2.4 In-silico mutagenesis localizes the sequence elements driving relation predictions

**Figure 6:**
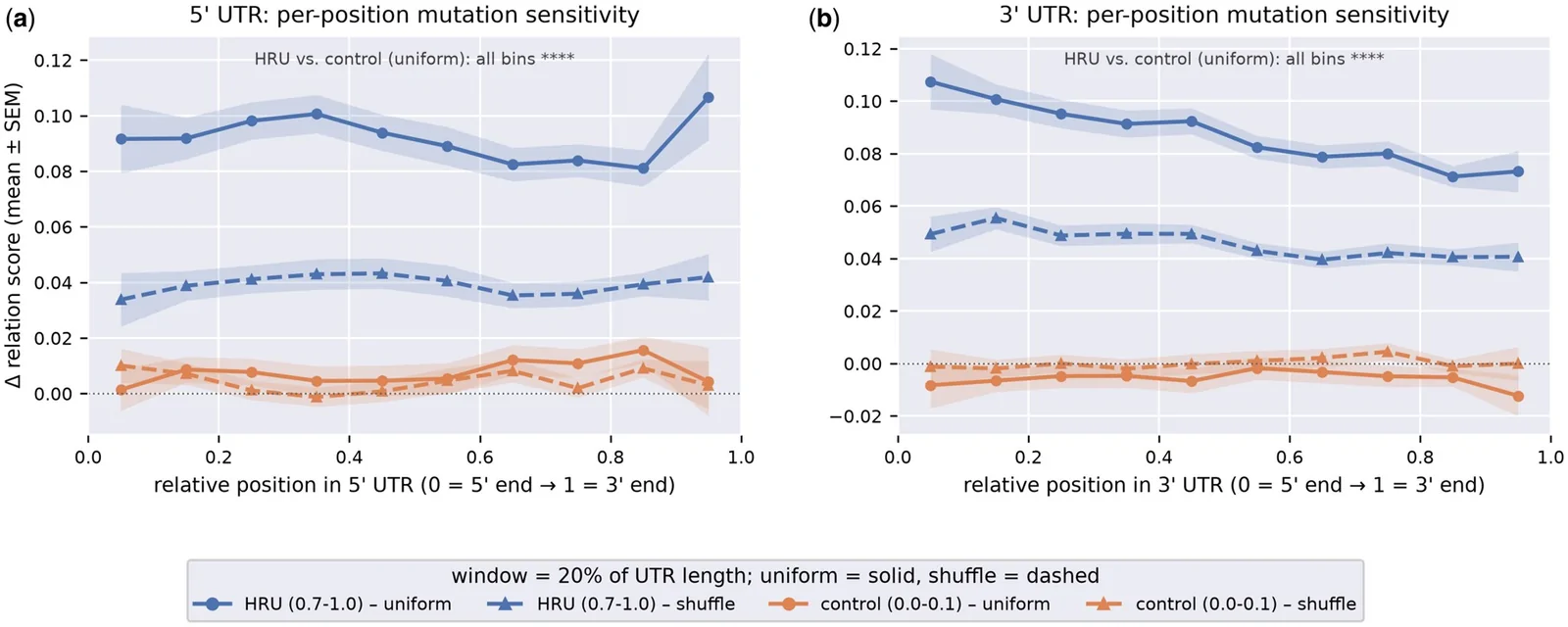
In-silico mutagenesis by sliding-window randomization of Highly Related UTRs (HRUs). A window (20% of the UTR length, 5% stride) was randomized while the partner UTR was kept intact, and the change in relation score (Δ= baseline − randomized, averaged over 10 trials) was measured along each UTR on the human dataset (contrastive learning with RiNALMo). (a,b) Mean ± SEM Δ across ten relative-position bins of the 5′ UTR (a) and 3′ UTR (b) for HRUs (blue; relation score 0.7–1.0) and randomly sampled control transcripts (orange; relation score 0.0–0.1), under uniform (solid lines) and composition-preserving shuffle (dashed lines) randomization. HRUs were significantly more sensitive than controls at every bin (Mann–Whitney U test with Benjamini–Hochberg correction; all bins FDR $<10^{-7}$ under uniform randomization). A window-size-robustness replicate using a 10% window is shown in Fig. S3, available as supplementary data at *Bioinformatics* online.

To understand what the model learns beyond pairing UTRs and how HRUs differ from weakly related pairs, we performed position-resolved in-silico mutagenesis on the 157 HRUs and on 157 control transcripts from the lowest relation-score bin (0.0–0.1). At every positional bin of both UTRs, HRUs were markedly more sensitive to randomization than controls (Mann–Whitney U test, Benjamini–Hochberg FDR $<10^{-7}$ at every bin; minimum FDR $=7\times 10^{-27}$; Fig. 6a and b). The mean Δ of HRUs remained high across the 5′ and 3′ UTRs (≈0.08–0.11), whereas control transcripts showed essentially no sensitivity (mean Δ≈0 in the 5′ UTR and slightly negative in the 3′ UTR), corresponding to an order-of-magnitude difference in mean sensitivity. This indicates that the high relation scores of HRUs are not coincidental matches but are driven by specific, localizable sequence content that weakly related pairs lack.

The mutation sensitivity of individual HRUs was strongly localized. Across the 157 HRUs, the maximum-sensitivity Δ averaged 0.28 (*SD* 0.13), and eight transcripts exceeded the mean + 2 *SD* threshold (0.54) as outliers (Table 2), with the maximum-sensitivity window residing in the 5′ UTR for six of the eight. The most striking case was HIC1, for which randomizing a single 31-nt window in its 156-nt 5′ UTR collapsed the relation score from 0.695 to 0.012 (a 98% reduction). For the long 3′ UTR transcripts among the outliers (TGFB2, OR13G1), the sensitivity peaked near the 5′ end of the 3′ UTR (relative position 0.10–0.15, immediately downstream of the stop codon) rather than being uniformly distributed (Fig. S2, available as supplementary data at *Bioinformatics* online).

**Table 2:** The eight most mutation-sensitive Highly Related UTR (HRU) transcripts identified by sliding-window in-silico mutagenesis (uniform randomization; maximum-sensitivity window above the mean + 2 *SD* threshold).

Gene	Transcript	Region	Window (nt; rel.)	**Baseline ** → ** rand.**	Δ **(drop)**	Candidate element
HIC1	ENST00000619757.5	5′ UTR	16–47 (0.20)	0.695 → 0.012	0.684 (98%)	m^6^A (DRACH) in GC-rich structure
EPHA8	ENST00000166244.8	5′ UTR	118–147 (0.90)	0.761 → 0.163	0.598 (79%)	GC-rich, Kozak-proximal context
GZMM	ENST00000592501.5	5′ UTR	64–94 (0.52)	0.661 → 0.071	0.590 (89%)	Stem-loop secondary structure
UNC5A	ENST00000509580.2	5′ UTR	180–226 (0.88)	0.827 → 0.255	0.572 (69%)	GC/C-rich context
TGFB2	ENST00000366930.9	3′ UTR	0–651 (0.10)	0.769 → 0.198	0.571 (74%)	AU-rich element (AUUUA; HuR)
RBBP8NL	ENST00000252998.2	5′ UTR	130–163 (0.90)	0.768 → 0.208	0.561 (73%)	RNA G-quadruplex (rG4)
OR13G1	ENST00000642119.1	3′ UTR	65–326 (0.15)	0.749 → 0.192	0.556 (74%)	AU-rich 3′ UTR region
SLITRK3	ENST00000475390.2	5′ UTR	82–247 (0.20)	0.729 → 0.175	0.554 (76%)	Polypyrimidine tract (PTBP1)

Δ is the drop in relation score upon randomizing the indicated window (baseline − randomized; mean of 10 trials). Candidate elements are sequence-based assignments from a literature-consensus motif scan (Table S2); a composition-versus-structure decomposition of the same windows is provided in Supplementary Note S1 and Table S3, available as supplementary data at *Bioinformatics* online.

We next characterized the candidate sequence elements underlying these sensitive windows by scanning each window against literature-consensus RBP-binding and RNA-structural motifs (Table S2, available as supplementary data at *Bioinformatics* online) and by decomposing its sensitivity into composition- and structure-driven components (Supplementary Note S1 and Table S3, available as supplementary data at *Bioinformatics* online). The most sensitive windows coincided with recognized regulatory features: candidate structured 5′ UTR elements (an RNA G-quadruplex in RBBP8NL (Kumari *et al*. 2007, Dumas *et al*. 2021) and a stem-loop in GZMM), GC-rich, start-codon-proximal initiation contexts in EPHA8 and UNC5A, and two AUUUA AU-rich elements immediately downstream of the TGFB2 stop codon (Chen and Shyu 1995, Barreau *et al*. 2005), consistent with regulation by stability factors such as HuR/ELAVL1 (Brennan and Steitz 2001) and with TGFB2 being a known ARE-containing transcript. This localization suggests that the model grounds its predictions in known regulatory features rather than merely matching sequences. We note that the motif assignments are sequence-based candidates rather than experimentally confirmed sites, and that the structural contributions are inferred from the recovery of Δ under dinucleotide-preserving randomization rather than from direct folding.

## 4 Discussion

In this study, we investigated predictable relationships between the 5′ and 3′ UTRs within the same mRNA molecule. To explore this, we employed a deep learning-based approach that integrated sequence prediction with multi-faceted downstream analyses. In our paired prediction task, incorporating sequence embeddings from the RNA pre-training model RiNALMo with contrastive learning achieved the highest prediction accuracy. The strong performance of our predictive model suggests a non-negligible association between the 5′ UTR and 3′ UTR sequences.

Leveraging association scores from our prediction task, subsequent downstream analyses further revealed that mRNAs with highly related UTRs (HRUs) possess distinct sequence characteristics in terms of both length and MFE, along with different expression profiles compared to other mRNA populations. Notably, HRUs were enriched for transcripts encoding genes involved in brain development and neural function. HRU-containing mRNAs also exhibited a pronounced tendency to interact with RNA-binding proteins (RBPs) implicated in mRNA processing and showed significant variability in translation efficiency across cell types.

A position-resolved in-silico mutagenesis analysis further supported this interpretation at the level of individual elements. The sequence windows on which the model most relied to predict 5′–3′ UTR relatedness were not randomly distributed but coincided with recognized regulatory elements. Because HRUs were far more sensitive to such perturbations than weakly related transcripts at every position, these elements appear to be a genuine basis for the learned relationship rather than an artifact of sequence similarity. This element-level evidence complements our finding that HRUs harbor more stable 5′ UTR secondary structures, and it suggests that the coordination between 5′ and 3′ UTRs captured by the model is mediated by structured translational-control elements. Experimental validation of these candidate elements will be an important next step.

Although our findings indicate a clear association between 5′ and 3′ UTRs, the underlying molecular mechanisms remain elusive. We found that certain RBPs, which preferentially bind to HRUs, have gene ontologies commonly associated with mRNA processing in both 5′ and 3′ UTRs. This finding suggests that the interplay between UTRs may be involved in recruiting RBPs that mediate mRNA processing. For instance, in human p53 mRNA, RNPC1 is known to bind to both 5′ and 3′ UTRs, thereby inhibiting the association of the translation initiation factor eIF4E (Zhang *et al*. 2011). A similar mechanism may also occur in the HRUs we have found in this study.

Future investigations of the mechanistic basis of 5′–3′ UTR interactions may uncover novel regulatory pathways in mRNA translation. A deeper understanding of these processes could not only enhance our knowledge of post-transcriptional regulation but also contribute to the rational design of next-generation mRNA therapeutics.

## Supplementary Material

btag634_Supplementary_Data
