# Supplementary material for: Deciphering the comprehensive relationship between 5′ UTR and 3′ UTR sequences with deep learning
Source: Bioinformatics. 2026 Aug 26;42(9):btag634. doi: 10.1093/bioinformatics/btag634 (PMC13589785; doi:10.1093/bioinformatics/btag634)
Supplement: Supplementary file 1 [file btag634_supplementary_data.pdf]

Supplementary materials for "Deciphering the comprehensive relationship between 5′ UTR and 3′ UTR sequences with deep learning"

Kanta Suga, Keisuke Yamada and Michiaki Hamada

1 Supplementary Figures and Tables

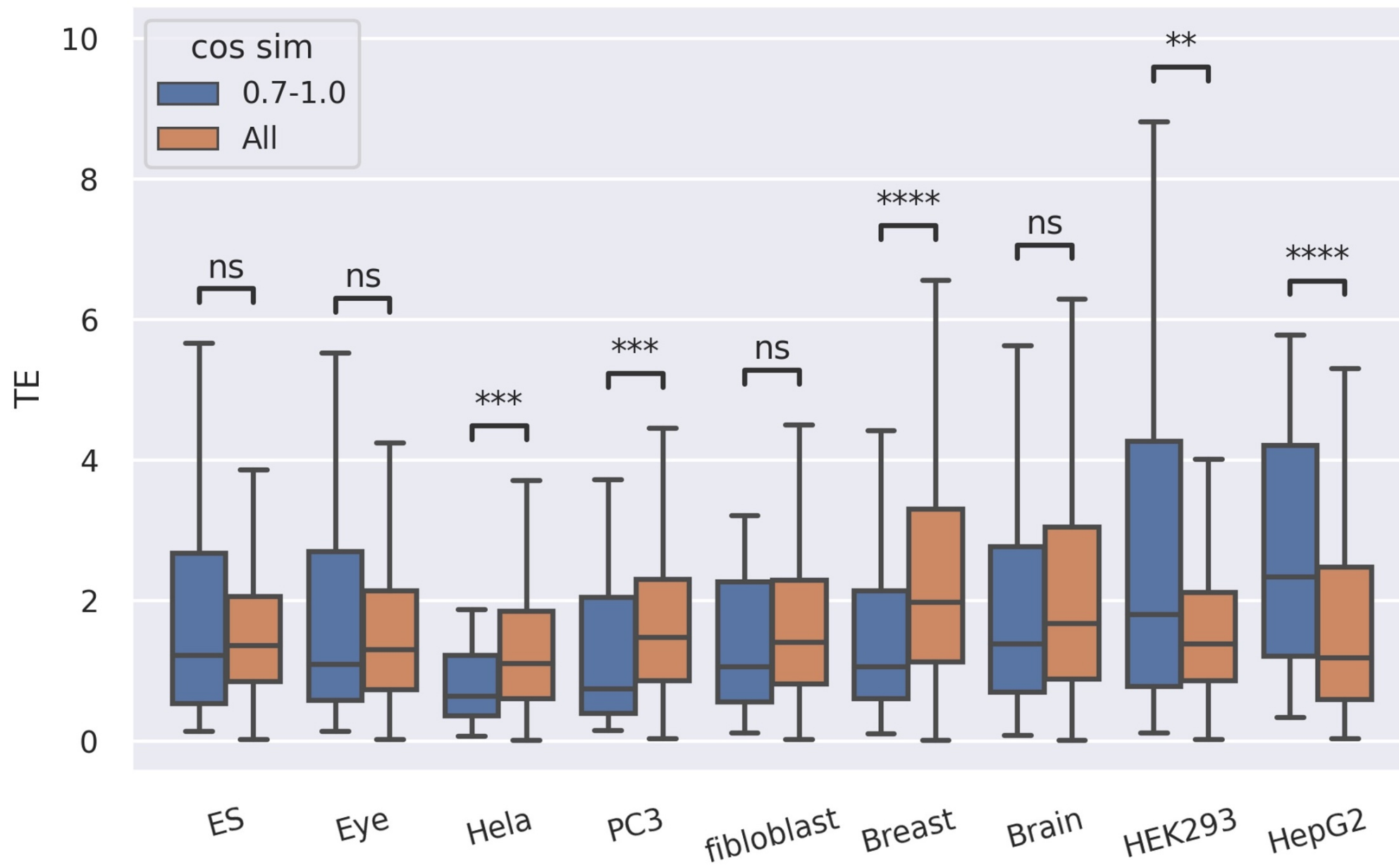

Fig. S1: Comparison of translation efficiency (TE) between HRUs and other mRNAs across 9 kinds of cell lines/tissue by the Mann-Whitney U test. TE was analyzed separately for each cell line/tissue

Table S1: A list of SRR-ID used for TE comparison analysis

SRR ID	Cell line/Tissue	Sequence type	Reference
SRR1562539	Brain	ribo-seq	Gonzalez *et al.* (2014)
SRR1562544	Brain	rna-seq	
SRR2075925	HEK293	ribo-seq	Iwasaki *et al.* (2016)
SRR2075930	HEK293	rna-seq	
SRR057526	HeLa	ribo-seq	Guo *et al.* (2010)
SRR057527	HeLa	rna-seq	
SRR403883	PC3	ribo-seq	Hsieh *et al.* (2012)
SRR403882	PC3	rna-seq	
SRR2064024	fibroblast	rna-seq	Tirosh *et al.* (2015)
SRR2064017	fibroblast	ribo-seq	
SRR1610260	ES	rna-seq	Werner *et al.* (2015)
SRR1610244	ES	ribo-seq	
SRR1573941	Breast	rna-seq	Rubio *et al.* (2014)
SRR1573939	Breast	ribo-seq	
SRR1976453	Eye	rna-seq	Tanenbaum *et al.* (2015)
SRR1976447	Eye	ribo-seq	
SRR8135336	HepG2	ribo-seq	Huang *et al.* (2019)
SRR8131644	HepG2	rna-seq	

Supplementary Note S1: composition-versus-structure decomposition

Each randomization scheme destroys the sequence content of a window while preserving a progressively larger, nested set of its statistical properties: *uniform* preserves none, *shuffle* preserves the base composition (mononucleotide

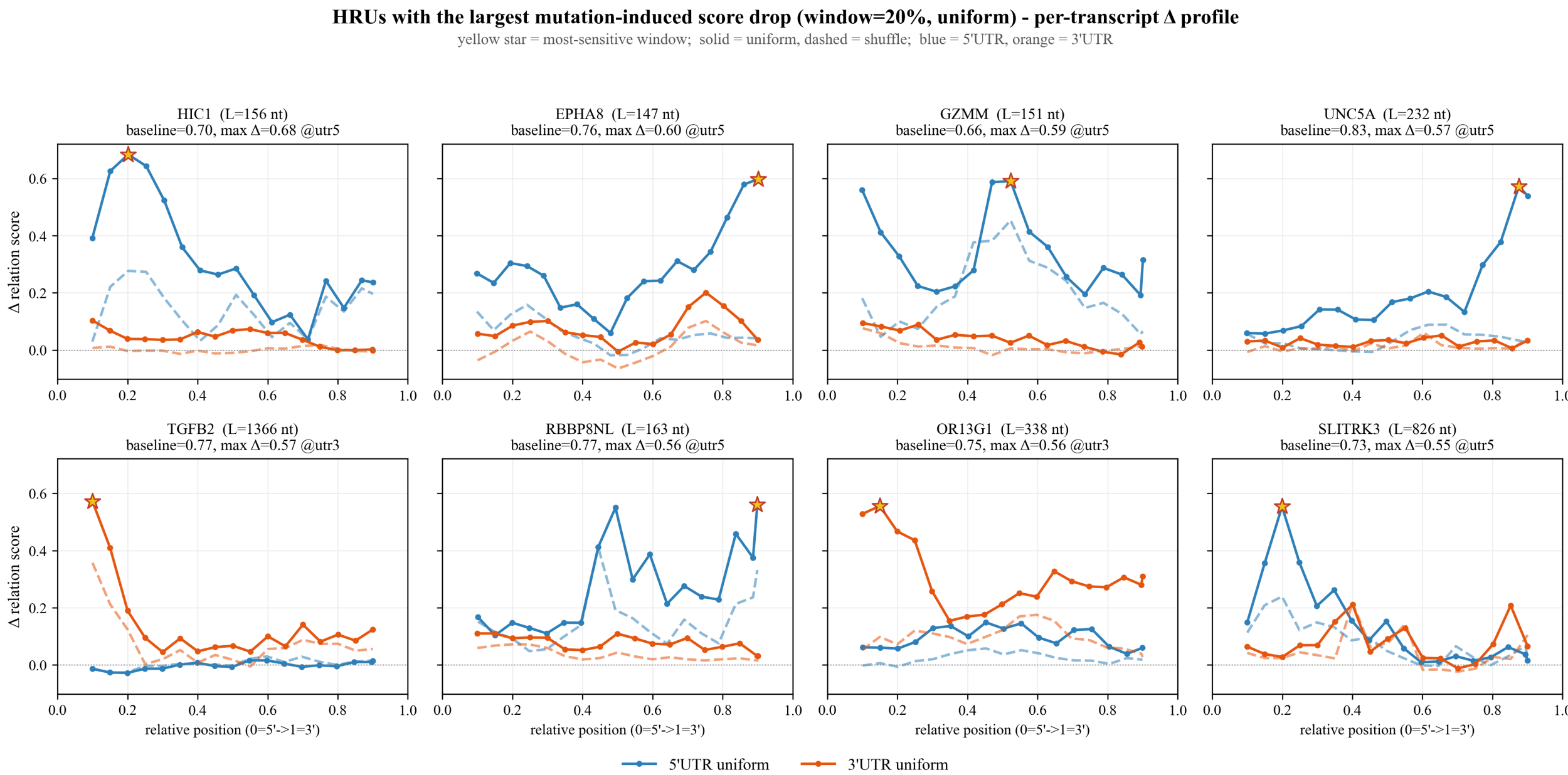

Fig. S2: **Per-transcript Δ profiles of the eight most mutation-sensitive HRUs.** For each transcript, Δ (the drop in relation score upon window randomization) is plotted against relative position for the 5′ UTR (blue) and 3′ UTR (orange) under uniform (solid) and shuffle (dashed) randomization; the star marks the maximum-sensitivity window. In each panel title, L is the length of the 5′ UTR of that transcript; the 5′ and 3′ UTRs are each plotted on their own relative-position axis (0–1), so the randomized window always spans 20% of the length of the UTR being scanned (not of L). See Methods and main-text Fig. 6 / Table 2.

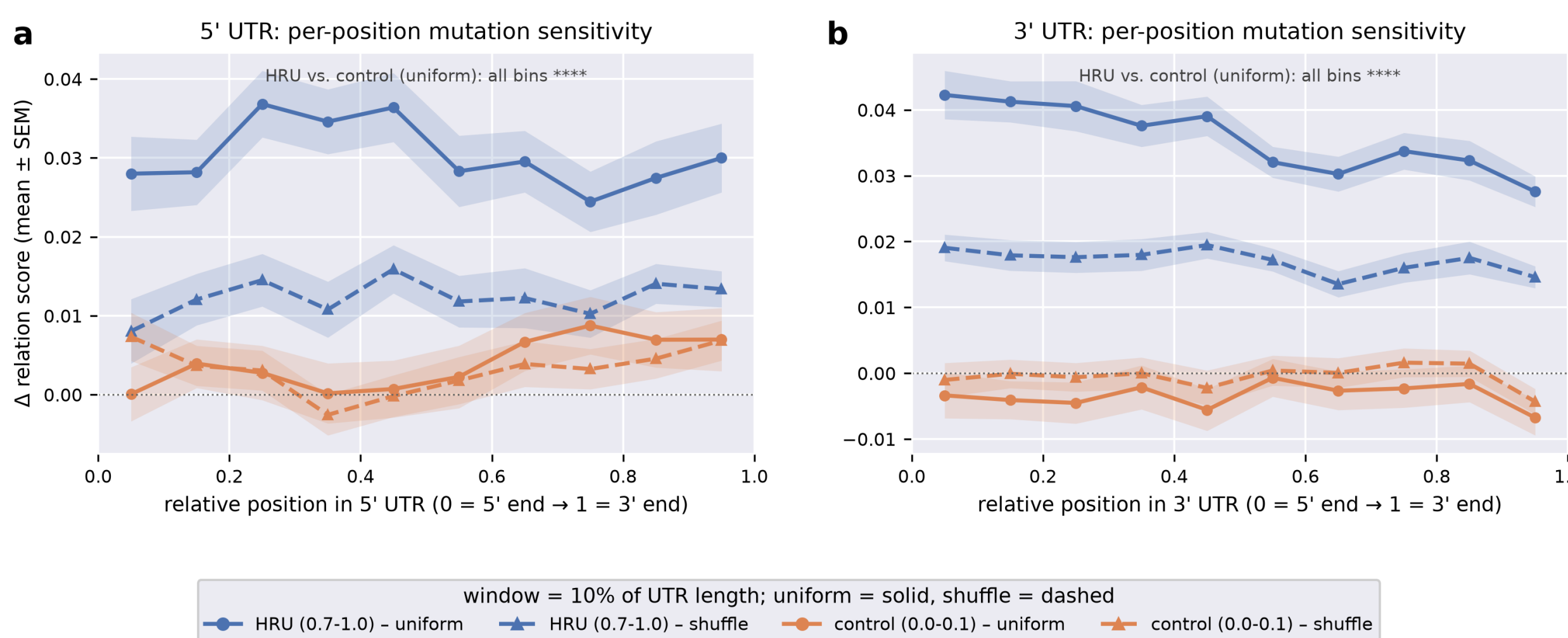

Fig. S3: **Window-size-robustness replicate of the position-resolved in-silico mutagenesis (window = 10% of the UTR length).** As in main-text Fig. 6 but with a 10% window (5% stride). Mean ± SEM Δ relation score (baseline − randomized) across ten relative-position bins of the 5′ UTR (a) and 3′ UTR (b) for HRUs (blue; relation score 0.7–1.0) and randomly sampled control transcripts (orange; relation score 0.0–0.1), under uniform (solid lines) and shuffle (dashed lines) randomization. The HRU-specific, position-localized sensitivity seen at the 20% window is reproduced at this finer window size.

Table S2: Sequence and motif characterization of the maximum-sensitivity windows of the eight outlier HRUs (main-text Table 2). GC is the GC content (%) of the window; "Context" notes structural positioning; motif hits are literature-consensus RBP-binding and RNA-structural patterns (counts in parentheses). For long 3′ UTR windows, multiple hits reflect a broad region rather than a single site (dilution effect).

Gene	Region	Window (nt)	GC (%)	Context	Motif hits (count)
HIC1	5′ UTR	16–47	71	—	m^6A DRACH/RRACH (×2); SRSF1 ESE (×1); G-run (×1)
EPHA8	5′ UTR	118–147	100	upstream of CDS (Kozak, −3 =G)	hnRNPK poly-C (×1)
GZMM	5′ UTR	64–94	73	—	G-run (×1)
UNC5A	5′ UTR	180–226	96	—	hnRNPK poly-C (×1); G-run (×1)
TGFB2	3′ UTR	0–651	35	immediately after stop codon	HuR/ELAVL1 ARE AUUUA (×2); ARE core WUAUUUAUW (×1); PTBP1 CU-rich (×3); poly-pyrimidine (×2); poly-U/TIA1 (×3); m^6A DRACH (×18); CPEB CPE (×1); SRSF1 ESE (×1)
RBBP8NL	5′ UTR	130–163	82	upstream of CDS (Kozak, −3 =G)	**G-quadruplex rG4 (×1)**; G-run (×1)
OR13G1	3′ UTR	65–326	32	—	HuR/ELAVL1 ARE AUUUA (×1); PUM1/2 PRE UGUANAUA (×1); PTBP1 CU-rich (×3); poly-pyrimidine (×2); poly-U/TIA1 (×1); m^6A DRACH (×1)
SLITRK3	5′ UTR	82–247	56	—	PTBP1 CU-rich (×3); poly-pyrimidine (×5); m^6A DRACH (×1); hnRNPK poly-C (×5)

frequencies), and *dinucleotide* preserves both the base composition and the dinucleotide frequencies. Because the nearest-neighbor (stacking) free energies that determine duplex stability are parameterized at the dinucleotide level (Turner and Mathews, 2010; Lorenz *et al.*, 2011), preserving dinucleotide frequencies approximately preserves the local base-pairing (secondary-structure) propensity of the window.

Scheme	Base composition	Dinucleotide freq. (pairing potential)	Higher-order order
uniform	destroyed	destroyed	destroyed
shuffle	preserved	destroyed	destroyed
dinucleotide	preserved	preserved	destroyed

The sensitivity Δ that survives a scheme therefore reflects only the properties that scheme preserves, and the difference in Δ between two adjacent schemes isolates the contribution of the single property that distinguishes them. Writing Δ_{uniform}, Δ_{shuffle} and Δ_{dinuc} for the Δ of the maximum-sensitivity window under the three schemes, we partition the total (uniform) sensitivity into three additive fractions that sum to one: a base-composition term $(\Delta_{\text{uniform}} - \Delta_{\text{shuffle}})/\Delta_{\text{uniform}}$, recovered when composition is held fixed; a base-pairing/secondary-structure term $(\Delta_{\text{shuffle}} - \Delta_{\text{dinuc}})/\Delta_{\text{uniform}}$, additionally recovered when dinucleotide frequencies are held fixed; and a single-stranded-order term $\Delta_{\text{dinuc}}/\Delta_{\text{uniform}}$, the residual sensitivity to higher-order arrangement.

Because the ordering $\Delta_{\text{uniform}} \geq \Delta_{\text{shuffle}} \geq \Delta_{\text{dinuc}}$ holds only in expectation, an individual term can be slightly negative under sampling noise (e.g. the base-pairing term of SLITRK3 in Supplementary Table S3), which we interpret as a negligible contribution from the corresponding property. The base-pairing term is an indirect, sequence-based indicator of secondary-structure potential rather than direct evidence of folding.

Table S3: Composition-versus-structure decomposition of the maximum-sensitivity window of each of the eight outlier HRUs (main-text Table 2). Δ_{uniform}, Δ_{shuffle} and Δ_{dinuc} are the drop in relation score (baseline − randomized; mean of 10 trials) under the three randomization schemes. Comp., BP and SS are the base-composition, base-pairing (secondary-structure) and single-stranded-order contributions, expressed as a percentage of the uniform Δ (Supplementary Note S1). Comp.+BP+SS= 100 % by construction; the negative BP for SLITRK3 reflects sampling noise around zero.

Gene	Region	Window (nt)	Δ_{uniform}	Δ_{shuffle}	Δ_{dinuc}	Comp.	BP	SS
HIC1	5′ UTR	16–47	0.684	0.277	0.101	59	26	15
EPHA8	5′ UTR	118–147	0.598	0.040	0.040	93	0	7
GZMM	5′ UTR	64–94	0.590	0.453	0.097	23	60	17
UNC5A	5′ UTR	180–226	0.572	0.033	0.026	94	1	5
TGFB2	3′ UTR	0–651	0.571	0.357	0.171	38	32	30
RBBP8NL	5′ UTR	130–163	0.561	0.332	0.052	41	50	9
OR13G1	3′ UTR	65–326	0.556	0.098	0.054	82	8	10
SLITRK3	5′ UTR	82–247	0.554	0.239	0.250	57	−2	45

References

Gonzalez, C. *et al.* (2014). Ribosome profiling reveals a cell-type-specific translational landscape in brain tumors. *Journal of Neuroscience*, **34**(33), 10924–10936.

Guo, H. *et al.* (2010). Mammalian micrornas predominantly act to decrease target mrna levels. *Nature*, **466**(7308), 835–840.

Hsieh, A. C. *et al.* (2012). The translational landscape of mtor signalling steers cancer initiation and metastasis. *Nature*, **485**(7396), 55–61.

Huang, H. *et al.* (2019). Histone h3 trimethylation at lysine 36 guides m6a rna modification co-transcriptionally. *Nature*, **567**(7748), 414–419.

Iwasaki, S. *et al.* (2016). Rocaglates convert dead-box protein eif4a into a sequence-selective translational repressor. *Nature*, **534**(7608), 558–561.

Lorenz, R. *et al.* (2011). Viennarna package 2.0. *Algorithms for molecular biology*, **6**, 1–14.

Rubio, C. A. *et al.* (2014). Transcriptome-wide characterization of the eif4a signature highlights plasticity in translation regulation. *Genome biology*, **15**, 1–19.

Tanenbaum, M. E. *et al.* (2015). Regulation of mrna translation during mitosis. *Elife*, **4**, e07957.

Tirosh, O. *et al.* (2015). The transcription and translation landscapes during human cytomegalovirus infection reveal novel host-pathogen interactions. *PLoS pathogens*, **11**(11), e1005288.

Turner, D. H. and Mathews, D. H. (2010). Nndb: the nearest neighbor parameter database for predicting stability of nucleic acid secondary structure. *Nucleic acids research*, **38**(suppl_1), D280–D282.

Werner, A. *et al.* (2015). Cell-fate determination by ubiquitin-dependent regulation of translation. *Nature*, **525**(7570), 523–527.
